# Concentrated Protein Body Product Derived from Rice Endosperm as an Oral Tolerogen for Allergen-Specific Immunotherapy—A New Mucosal Vaccine Formulation against Japanese Cedar Pollen Allergy

**DOI:** 10.1371/journal.pone.0120209

**Published:** 2015-03-16

**Authors:** Yuhya Wakasa, Hidenori Takagi, Nobumasa Watanabe, Noriko Kitamura, Yoshihiro Fujiwara, Yuko Ogo, Shimpei Hayashi, Lijun Yang, Masaru Ohta, Wai Wai Thet Tin, Kenji Sekikawa, Makoto Takano, Kenjirou Ozawa, Takachika Hiroi, Fumio Takaiwa

**Affiliations:** 1 Genetically Modified Organism Research Center, National Institute of Agrobiological Sciences, Kannondai, Tsukuba, Ibaraki, Japan; 2 Department of Allergy and Immunology, Tokyo Metropolitan Institute of Medical Science, Kamikitazawa, Setagaya-ku, Tokyo, Japan; 3 PrevenTec, Inc., Owashi, Tsukuba, Ibaraki, Japan; Wuhan Botanical Garden, Chinese Academy of Sciences, CHINA

## Abstract

The endoplasmic reticulum-derived type-I protein body (PB-I) from rice endosperm cells is an ideal candidate formulation for the oral delivery of bioencapsulated peptides as tolerogens for allergen-specific immunotherapy. In the present study, PBs containing the deconstructed Japanese cedar pollen allergens *Cryptomeria japonica 1* (Cry j 1) and Cry j 2 were concentrated by treatment with thermostable α-amylase at 90°C to remove the starch from milled rice powder, which resulted in a 12.5-fold reduction of dry weight compared to the starting material. The modified Cry j 1 and Cry j 2 antigens in this concentrated PB product were more resistant to enzymatic digestion than those in the milled seed powder despite the absence of intact cell wall and starch, and remained stable for at least 10 months at room temperature without detectable loss or degradation. The high resistance of these allergens could be attributed to changes in protein physicochemical properties induced by the high temperature concentration process, as suggested by the decreased solubility of the antigens and seed proteins in PBs in step-wise-extraction experiments. Confocal microscopy showed that the morphology of antigen-containing PB-Is was preserved in the concentrated PB product. The concentrated PB product induced specific immune tolerance against Cry j 1 and Cry j 2 in mice when orally administered, supporting its potential use as a novel oral tolerogen formulation.

## Introduction

Allergen-specific immunotherapy induces immunological tolerance to an allergen, reducing the clinical symptoms caused by IgE-mediated type-I allergy. Conventional allergy vaccines consist of crude allergens and are usually composed of a heterologous mixture of allergenic and nonallergenic compounds that can cause anaphylactic shock. Vaccination is achieved by administration of increasing doses of allergen extract during a period of 3 to 5 years through subcutaneous or sublingual routes. In a previous study, we used hypoallergenic derivatives to develop a rice seed-based vaccine for the safe and convenient treatment of aeroallergen disease such as that caused by Japanese cedar pollen, birch pollen, and house dust [1]. The tertiary structure of the native allergens required for IgE binding was altered by fragmentation, shuffling, or mutation, and these structurally deconstructed allergens were specifically expressed in transgenic rice endosperm [2–6]. Rice seed-based oral vaccination has several advantages in terms of safety, stability, and convenience compared with the conventional system based on subcutaneous injection of crude allergen extracts [7]. Recombinant proteins produced in rice show high stability at room temperature for several years, easy control of production scale, no contamination with mammalian pathogens, and are cost-effective. Furthermore, oral (needle-free) administration can reduce injection-associated pain.

Rice is an efficient bioreactor for the production of recombinant protein in terms of high biomass yield, low risk of gene flow due to self-pollination and ease of transformation. Furthermore, systems for the cultivation, harvesting, processing, and storage of rice are well established worldwide. Strategies for the accumulation of recombinant proteins in the seed have recently been developed in many laboratories [8–12]. Rice endosperm cells have two types of organelles for the accumulation of seed storage proteins, namely endoplasmic reticulum (ER)-derived protein bodies (PB-I) and protein storage vacuoles (PB-II), which are distinct in size and morphology [13, 14]. PB-I accumulates prolamins, whereas glutelins and α-globulin are deposited in PB-II. PBs are an attractive formulation for the delivery of recombinant proteins to gut-associated lymphoid tissue (GALT) as an oral vaccine, because encapsulation in PBs increases their resistance to harsh conditions such as low pH and proteolytic enzymes in the gastrointestinal tract. This was shown previously for proteins encapsulated in PB-I [15]. The use of PBs for the delivery of rice-based vaccines is therefore a reliable system for oral administration.

We recently generated transgenic rice in which the modified Japanese cedar major pollen allergens, *Cryptomeria japonica 1* (Cry j 1) and Cry j 2, were specifically produced in seeds. Cry j 1 was divided into three overlapping fragments that were expressed as fusion proteins with seed storage glutelins (GluA2, GluB1, and GluC). Cry j 2 was deconstructed by shuffling and was expressed as a secretory protein by attaching an N-terminal signal peptide and C-terminal ER retention signal (Lys-Asp-Glu-Leu). Modification of allergen proteins, such as that achieved by fragmentation and shuffling, decreases the binding activity to specific IgE antibodies [6]. These recombinant proteins are deposited in protein body-I (PB-I) together with cysteine-rich prolamins through the formation of intermolecular disulfide bonds in rice endosperm cells [16]. When transgenic rice seeds accumulating modified Cry j 1 and Cry j 2 were orally fed to model mice, suppression of allergen-specific IgE and effector cells and alleviation of clinical symptoms were induced by allergen-specific immune tolerance [6]. This transgenic rice seed is being developed without purification for use as an allergy vaccine under the guidance of the Ministry of Health, Labor, and Welfare of Japan.

Cooked rice is an ideal formulation as oral tolerogen for allergen-specific immunotherapy because of its low production cost and because it does not require expensive product purification (processing downstream of extraction and purification). However, as a final pharmaceutical formulation, the stock control and distribution of packed cooked rice presents challenges because of its large volume compared to pharmaceutical products based on tablets or capsules. To overcome this problem, we explored a new concentrated PB product formulation in which starch occupying more than 80% of grain weight was removed. This concentrated PB system can deliver sufficient amounts of antigen in the form of capsules or tablets and the processing is cost-effective. We showed that the quality and quantity of the recombinant proteins in the concentrated PB product were preserved, with minimal loss during processing, and their ability to induce immune tolerance by the oral route was demonstrated.

## Materials and Methods

### Plant Materials

Modified forms of the major Japanese cedar pollen allergens, *Cryptomeria japonica 1* (Cry j 1) and *Cryptomeria japonica 2* (Cry j 2), which were altered by fragmentation and shuffling, respectively, were expressed in the edible part (endosperm) of rice seeds under the control of rice endosperm-specific promoters [17]. The rice (*Oryza sativa* L.) cultivar ‘Koshihikari’ variety a123 and T5–T7 seeds were used in this study.

### Concentration of the Protein Body Fraction from Rice Seeds

Milled transgenic rice seeds were prepared by adding 150 mL of hot water (at approximately 90°C) and 1 mL of heat-resistant α-amylase Termamyl120L (Novozymes, Bagsvaerd, Denmark) to 75 g of milled rice powder in a 500 mL tube. After mixing, tubes were incubated at 90°C for 1 h to digest the seed starch. The mixture was centrifuged at 5000 rpm for 10 min at room temperature and the supernatant was discarded. The pellet was washed with hot water. Centrifugation and washing steps were repeated three times. Subsequently, the pellet (concentrated PB product) was spread onto a dish and dried at 60°C overnight in an incubator. The dried protein body fraction was ground into a fine powder and used as concentrated PB product.

### Immunoblotting

Mature seeds were individually ground into a fine powder with a multi-bead shocker (YASUI KIKAI, Tokyo, Japan). For total protein extraction, 500 μL of total protein extraction buffer [50 mM Tris-HCl pH 6.8, 8 M urea, 4% SDS, 20% glycerol, 5% 2-mercaptoethanol, 0.01% bromophenol blue] were added to seed powder (12.5 mg) or concentrated PB product (1 mg) and vortexed for 1 h at room temperature. The mixture was centrifuged at 14,000 rpm for 20 min at room temperature. Two-micro liters of protein extracts were separated by SDS-PAGE and proteins were blotted onto Immobilon-P PVDF transfer membranes (Millipore, Billerica, MA). Membranes were blocked in 5% skim milk for 1 h and reacted with primary antibody (1:10000 dilution) at 4°C for 16 h, followed by secondary anti-rabbit IgG conjugated HRP antibody at a 1:10000 dilution for 3 h at room temperature. Signals were detected with the Clarity Western ECL substrate (BIO-RAD, Hercules, CA).

### ELISA

Proteins were extracted from 50 mg rice of seed powder or 25 mg of concentrated PB product with 1% SDS, 50 mM 2-mercaptoethanpl, 50 mM Tris pH8.0, and 0.5 M NaCl using a sonicator. Proteins were precipitated with ethanol and dissolved with 6 M guanidine-HCl, 50 mM Tris pH8.0, and 50 mM 2-mercaptoethanol. Sample and standard proteins were diluted with the same buffer and put in ELISA plates. Antibody reaction and colorization were performed according to a conventional indirect ELISA method [18]. Monoclonal anti-Cry j 1 and anti-Cry j 2 antibodies, and polyclonal anti- Cry j 1 F3 peptide antibody were used as primary antibodies, and anti-mouse or anti- rabbit goat IgG conjugated with alkaline phosphatase (ALP) was used as secondary antibody. An ALP development kit (KPL pNPP phosphatase substrate system 50–80–00; KPL, Gaithersburg, MD) was used for color development.

### Protein, Lipid, Carbohydrate, Moisture, and Ash Contents of Seed Powder and Concentrated PB Product

Measurements of these components were performed by a commercial service provider (Japan Food Research Laboratories, Tokyo, Japan).

### Confocal microscopy

Milled rice powder or concentrated PB product (10 mg) were used for confocal microscopy. After immuno-staining of samples, rhodamine B was used to stain PB-I. Samples were spread on glass slides and sealed with a cover glass. The samples were observed with a confocal laser scanning microscope (FLUOVEIW; OLYMPUS, Tokyo, Japan).

### *In Vitro* Digestion Experiment

For pepsin digestions, 10 mg of seed powder or 1 mg of concentrated PB product powder was dissolved in 300 μL of reaction buffer (30 mM sodium chloride, 0.1% pepsin at pH 1.2) and incubated at 37°C for 0, 0.5, 1, 3, 5, 15, 30, 60, and 120 min. After incubation, 100 μL of reaction stop solution (160 mM Na_2_CO_3_) and 800 μL of protein extraction buffer were immediately added to the reaction mixtures. Mixtures were vortexed for 1 h and centrifuged at 13,000 × *g* for 10 min after boiling for 5 min to inactivate enzymes. Six microliters of sample was subjected to SDS-PAGE and immunoblot analyses. As control proteins, total seed proteins from transgenic rice seed were used. Total seed proteins were purified by chloroform methanol precipitation [19].

### Step-Wise Protein Extraction

In the first step, globulin was extracted from 40 mg of seed powder or 4 mg of concentrated PB product with 600 μL saline buffer (0.5 M NaCl in 10 mM Tris-HCl, pH 6.8). In the second step, endogenous glutelins were extracted with 600 μL 1% (v/v) lactic acid, followed by extraction of cysteine-poor and cysteine-rich prolamins with 600 μL 60% (v/v) *n*-propanol containing 5% (v/v) 2-mercaptoethanol. Finally, recombinant proteins were extracted with 600 μL 1% lactic acid. Each extraction step was accomplished by re-suspending seed powder or concentrated PB product in the solution and sonicating on ice for 2 min. After centrifugation (14,000 rpm for 10 min at room temperature), the pellets were washed twice with the same solution.

### Antibodies

Anti-GluA fused Cry j 1 F1, anti-GluB fused Cry j 1 F2, anti-GluC fused Cry j 1 F3, and anti-shuffled Cry j 2 monoclonal mouse antibodies were produced by the hybridoma technique. Anti-GluB, anti-RM1, anti-RM2, anti-10 kDa prolamin, and anti-16 kDa prolamin rabbit polyclonal antibodies were produced against synthetic peptides as peptide antibodies [20, 21]. An anti-α-amylase rabbit polyclonal antibody was produced against purified α-amylase Termamyl 120L. The specificity of these antibodies against target proteins was verified prior to their use.

### Analysis of the Uptake of Recombinant Proteins into Ileum and Jejunum Cells in Mice

Balb/c mice were intragastrically administered with 100 mg of GFP-fused shuffled Cry j 2 rice seed powder (S5 Fig.) suspended in 1 mL of PBS or wild-type rice. After 4, 8 and 12 h, intestinal samples were dissected, fixed with 4% formaldehyde solution for 3 h at room temperature, and embedded in Tissue-Tek OCT compound (Sakura Finetek, Tokyo Japan). Frozen sections (7 μm) were mounted in VECTRASHILD mounting medium (VECTOR, Burlingame, CA) and examined with a fluorescence microscope (BZ-9000; Keyence, Osaka Japan).

### Feeding of Rice Seed or PB Condensation Product and Sensitization of Mice

All experiments were conducted in accordance with the Guidelines for the Care and Use of Animals published by the Tokyo Metropolitan Institute of Medical Science, and the experimental protocols were approved by the Animal Use and Care Committee of the Tokyo Metropolitan Institute of Medical Science. Six-week old BALB/c male mice were freely fed wild-type rice seed powder, transgenic rice seed powder, wild-type concentrated PB product, or transgenic concentrated PB product for 7 days from week 0 to week 1. The daily amounts of rice seed powder and concentrated PB product consumed by each mouse were approximately 2.3 g and 1.3 g, respectively. At week 1, mice were intraperitoneally sensitized with 0.1 mg total protein extract of Japanese cedar pollen (Cosmo Bio) adsorbed on 5 mg Alum (aluminum hydroxide, Cosmo Bio) containing recombinant mouse IL-4 (R&D Systems) at 0.1 μg per mouse to maximize the induction of allergen-specific IgE responses. At week 3 and week 5, mice were intraperitoneally boosted with 0.1 mg total protein extract of Japanese cedar pollen adsorbed on 5 mg Alum. At week 6, the levels of allergen-specific IgE and splenic T-cell responses were examined as described previously [6].

### Statistics

Data are presented as mean values ± standard deviation. Statistical significance was determined by Student’s *t*-test.

## Results

### Characterization of Transgenic Rice Seeds

**Fig 1:**
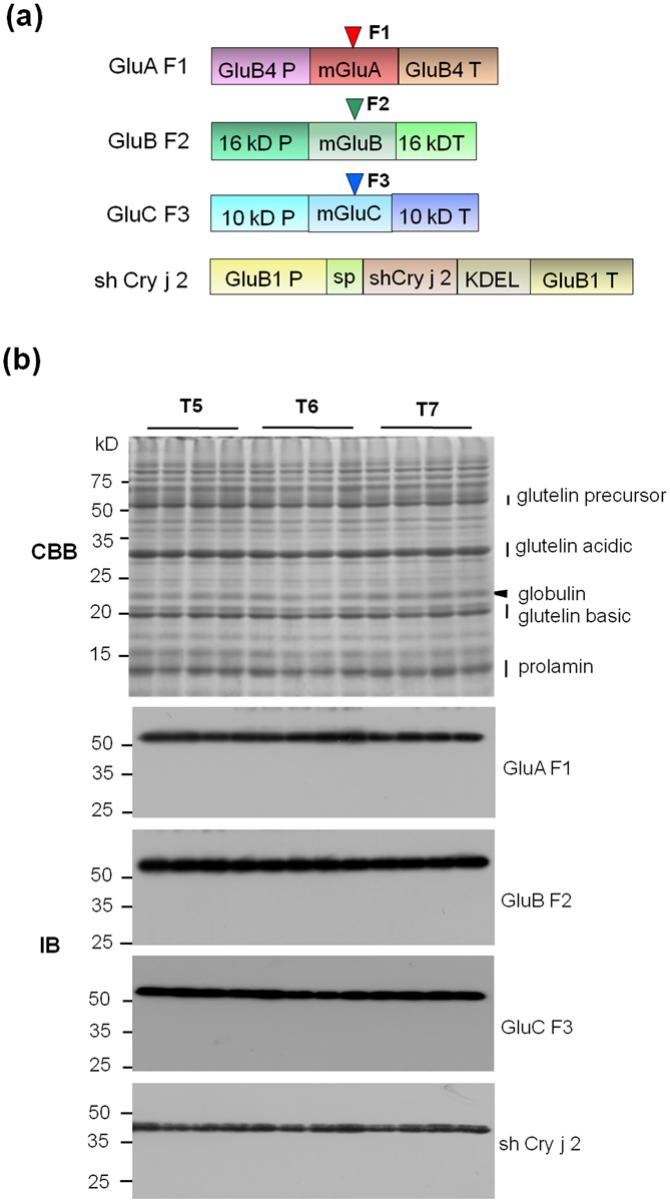
Transgenic rice seed used in the study. (a) Four gene cassettes expressed in transgenic rice seeds. GluB4 P, glutelin B4 promoter; 16 kD P, 16 kDa prolamin promoter; 10 kD P, 10 kDa prolamin promoter; GluB1 P, Glutelin B1 promoter; mGluA, modified glutelin A2 coding region; mGluB, modified glutelin B1 coding region; mGluC, modified glutelin C coding region; GluB4 T, glutelin B4 terminator; 16 kD T, 16 kDa prolamin terminator; 10 kD T, 10 kDa prolamin terminator; GluB1 T, glutelin B1 terminator; F1, Cry j 1 F1; F2, Cry j 1 F1; F3, Cry j 1 F3; shCry j 2, shuffled Cry j 2; SP, Glutelin B1 signal peptide; KDEL, ER retention signal. (b) SDS-PAGE (CBB) and immunoblot (IB) analyses of transgenic rice seed (T5–T7). The positions of glutelin precursor, glutelin acidic or basic, globulin, and prolamin on the SDS-PAGE gel are indicated on the right side of the panel. Immunoblot detection of transgene products derived from four gene cassettes.

Transgenic rice plants expressing the modified Japanese cedar pollen allergens, Cry j 1 and Cry j 2, were used in this study [6]. The four recombinant proteins (GluA F1, GluB F2, GluC F3, and shuffled Cry j 2, Fig. 1a) showed stable accumulation in the seeds of T5–T7 transgenic rice plants (Fig. 1b). These transgenic rice lines were used in subsequent experiments.

### Concentration of Protein Bodies (Pbs) from Milled Rice

**Fig 2:**
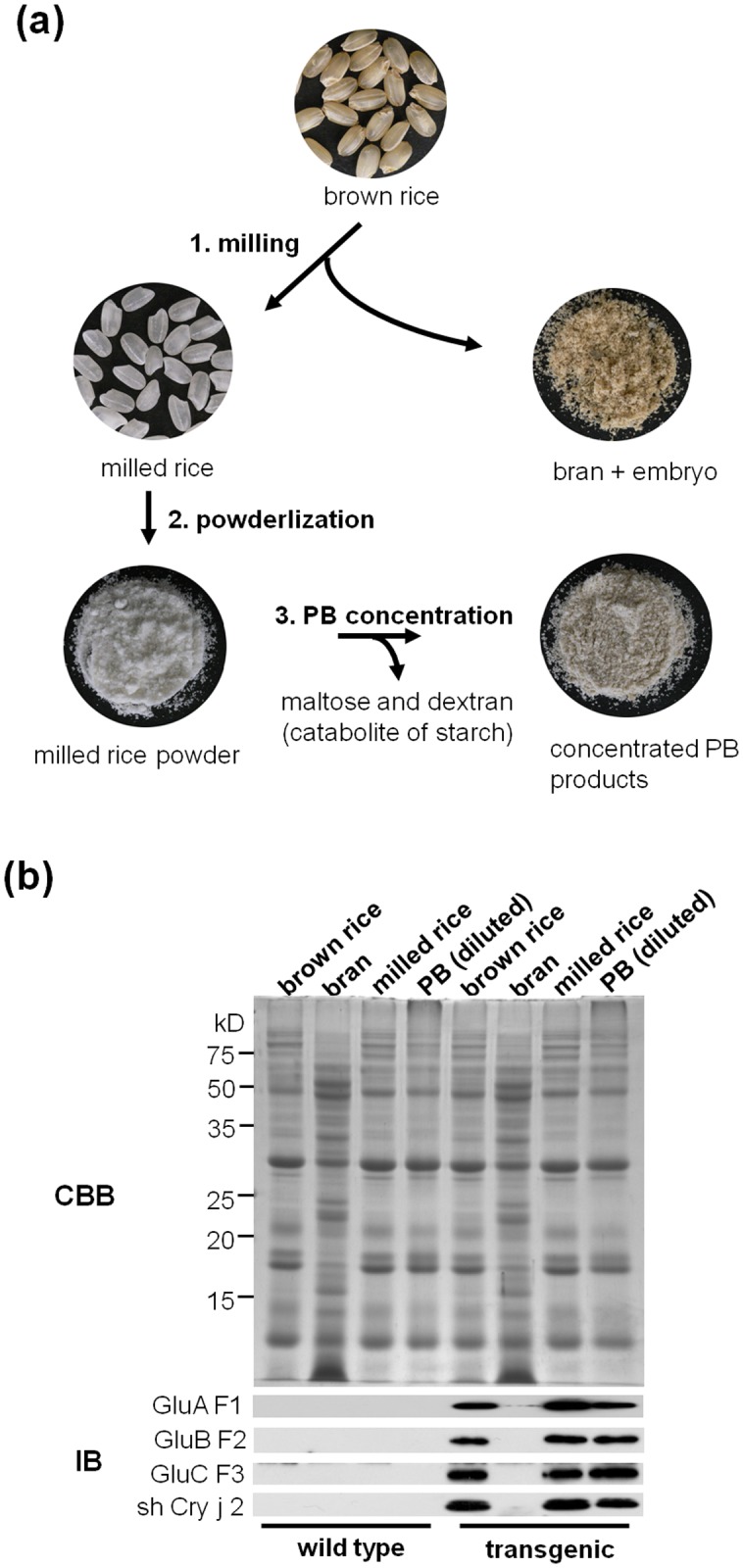
Schematic of the preparation of concentrated PB product. (a) Outline describing the preparation of concentrated PB product. (b) SDS-PAGE and immunoblot analyses of brown rice, bran, milled rice, and concentrated PB product in wild-type and transgenic rice seed. The amounts of seed proteins in the concentrated PB product were approximately 12-fold higher than those in milled rice; therefore, total protein extracts of concentrated PB products were diluted before SDS-PAGE.

A schematic describing the preparation of concentrated PB product is shown in Fig. 2a.

Hulled rice seeds were milled to remove the embryo and bran. Milled rice seeds were ground into a fine powder and suspended in a thermostable α-amylase (α-amylase Termamyl 120L) solution. The suspension was incubated at 90°C for starch gelatinization leading to smooth digestion of the starch by α-amylase. The saccharification liquid derived from the starch was discarded and the pellet, containing the concentrated PB product, was washed to completely remove the saccharification liquid. The concentrated PB product was then dried in an incubator at 60°C overnight and ground into a fine powder. Approximately 8.1 g of the concentrated PB product was produced from 100 g of milled seed powder (8.5 g, 8.0 g, and 7.8 g), representing an approximately 12.5-fold concentration.

### Characterization of Concentrated PB Product

The distribution of recombinant proteins among brown rice, bran, milled rice, and PB product was investigated by immunoblot analysis using specific antibodies (Fig. 2b). Before being subjected to SDS-PAGE, the deconstructed Cry j 1 and Cry j 2 proteins in the concentrated PB extract were diluted (12.5-fold) to adjust the quantity of loaded samples. The recombinant proteins were not detected in non-transgenic rice samples (Fig. 2b). Specific signals for the recombinant proteins were detected with equal intensity in brown rice, milled rice, and PB extract from transgenic rice seeds, whereas weak signals were detected in the bran fraction. This indicated that the recombinant proteins were specifically expressed in endosperm tissue under the control of the rice endosperm-specific promoters used in this study. Immunoblot analysis of the levels of endogenous seed storage proteins, including GluB (GluB1 and GluB2), 26 kDa α-globulin (Glb-1), 13 kDa cysteine rich prolamin (RM1), and 13 kDa cysteine-poor prolamin (RM2) in the same tissues (S1 Fig.) showed a pattern similar to that observed in the recombinant proteins, with high levels of expression in brown rice, milled rice, and PB extract, and low levels in the bran fraction (S1 Fig.).

Comparison of the protein content between the milled seed powder and the concentrated PB product showed that protein accounted for approximately 6.9% of the total weight in the milled seed powder, whereas it accounted for 72.6% in the PB product (Table 1).

**Table 1:** Component analysis of milled rice powder and concentrated PB product

	Protein (g/100 g)	Lipid (g/100 g)	Carbohydrate (g/100 g)	Moisture (g/100 g)	Ash (g/100 g)	GluA F1 mg/g	GluB F2 mg/g	GluC F3 mg/g	Shuffled Cry j 2 mg/g
**Milled rice**	6.9	1.5	82.2	8.8	0.6	0.33	0.3	0.37	0.56
**Concentrated PB product**	72.6	4.7	15.3	4.0	3.4	2.84	2.03	2.93	3.67

The levels of four recombinant proteins were examined in milled rice and PB extract by ELISA (Table 1). The results showed that the concentrations of GluAF1, GluBF2, GluCF3, and shuffled Cry j 2 were 2.84, 2.03, 2.93, and 3.67 mg/g, respectively, in the PB product, representing 8.6-, 6.8-, 7.9-, and 6.6-fold concentrations, respectively, compared to the levels in milled rice. Immunoblotting and ELISA together with measurement of protein amounts suggested that a small amount of recombinant protein was lost during the amylase digestion and washing steps. Lipid, carbohydrate, moisture and ash contents were also investigated (Table 1). Lipid and ash contents were concentrated by approximately 3- and 6- fold, whereas moisture and carbohydrate were reduced from 8.8% to 4.0% and from 82.2% to 15.5%, respectively. The carbohydrate content in the PB product was 15.3%; however, this may have been derived from broken and punctured cell wall components such as cellulose and pectin rather than residual starch, because the PB product did not stain easily with iodine solution (S2 Fig.).

**Fig 3:**
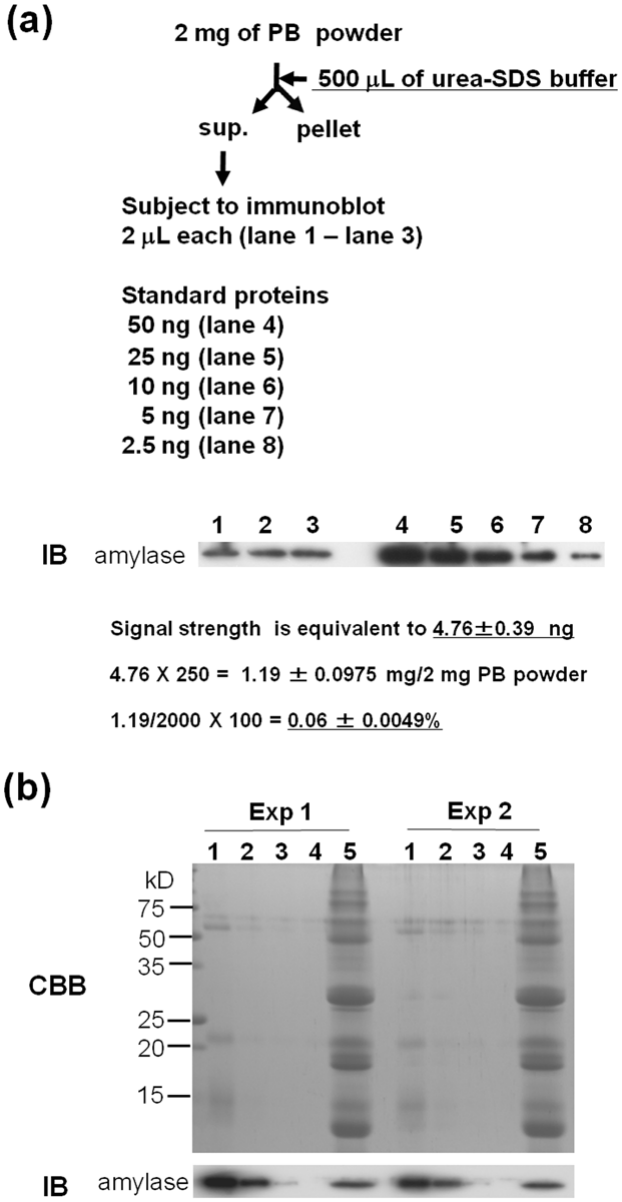
Detection of residual α-amylase in the concentrated PB product. (a) One-microliter of protein extract from 2 mg of concentrated PB product was subjected to immunoblot analysis (five independently prepared samples). The α-amylase level in each signal was estimated by comparison with standards (2.5, 5.0, 10, and 25 ng) of α-amylase purified from α-amylase Termamyl120L. The amount of residual α-amylase was calculated as a percentage. (b) Detection of residual α-amylase during the preparation of concentrated PB product. Supernatants obtained in the first centrifugation after α-amylase treatment (lane 1), second to fourth centrifugation after washing (lane 2 to lane 4) and protein extracts from the final pellet (concentrated PB product, lane 4) were analyzed by SDS-PAGE (CBB) and immunoblotting (IB). A volume of 2 μL of sample was subjected to SDS-PAGE. Experiments were repeated two times (Exp 1 and Exp 2).

The concentrated PB product consisted mainly of recombinant proteins and endogenous rice seed proteins, with small amounts of lipids, carbohydrate, moisture, and ash. The fact that most of these components are derived from the rice seed ensures the safety of the PB product. Risk assessment for chronic toxicity, immunotoxicity, and embryo-toxicity was performed by feeding rats 20 g of the concentrated PB product prepared from 250 g of milled rice powder/kg/rat for 7 days. No negative side-effects were observed and the safety of the PB product was confirmed. However, the concentrated PB product contained α-amylase, which is an exogenous additive. The digestion of rice starch was performed with thermostable α-amylase (Termamyl 120L, Novozymes) derived from *Bacillus licheniformis*, a food grade enzyme recommended by the FAO/WHO Joint Expert Committee on Food Additives (JECFA) and Food Chain Crisis Management Framework (FCC). The remaining level of α-amylase in the isolated PB product was analyzed by immunoblotting using an anti-α-amylase antibody. As shown in Fig. 3, the residual α-amylase in the concentrated PB product accounted for approximately 0.06% of the total weight.

The denaturation of the enzyme during the 90°C/1 h incubation could have affected its solubility, possibly explaining the residual α-amylase of the final PB product (S3 Fig.).

The active form of α-amylase Termamyl 120L forms a strong high molecular weight complex that is water soluble. The monomeric form (approximately 55 kDa, arrowhead) is inactive and water insoluble. The high molecular weight complex of α-amylase showed lower affinity for an anti-amylase antibody than the monomeric form of the same protein.

Although most of the water soluble α-amylase was removed from the concentrated PB product by washing three times, a small amount of denatured enzyme was recovered in the pellet together with the PBs after centrifugation (Fig. 3b).

### Recombinant Protein in the Concentrated PB Product Shows High Stability During Long Term Storage at Room Temperature

**Fig 4:**
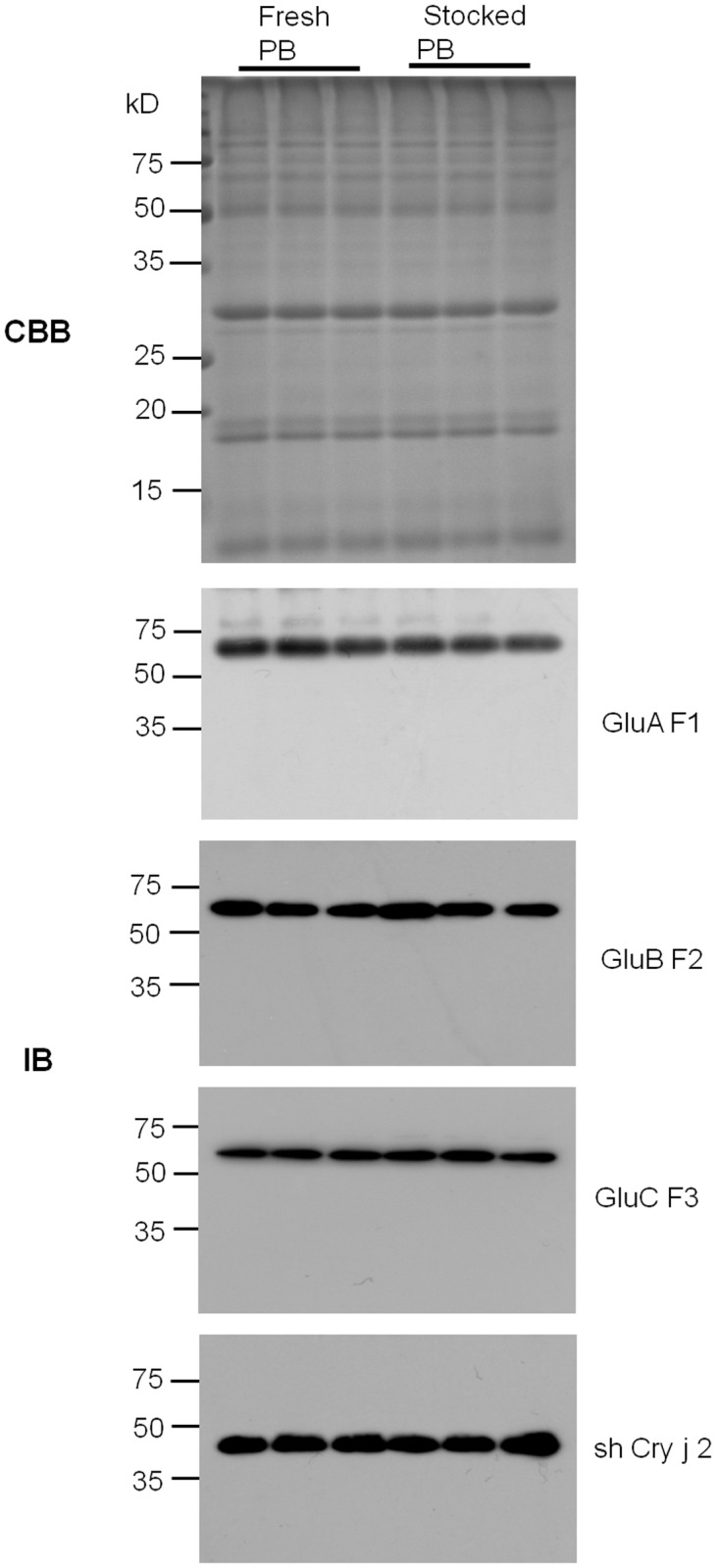
Evaluation of shelf life of the concentrated PB product. Concentrated PB products were prepared from transgenic rice seeds and stored at room temperature for 10 month (Stocked PB). Ten month after, concentrated PB products were prepared again from the same transgenic rice seeds (Fresh PB). Total proteins were extracted from flesh and stocked concentrated PB products at the same time and subject to SDS-PAGE (CBB) and immunoblot (IB) analyses.

The stability of recombinant proteins deposited in the PB product is important for the development of pharmaceuticals. The quality and quantity of the recombinant proteins in the concentrated PB product that was stored in a sealed container at room temperature for 10 months were compared by immunoblot analysis. No differences in the signal patterns of the recombinant proteins were observed between the fresh and stocked PB samples (Fig. 4).

Furthermore, no signals derived from degradation products of recombinant proteins were detected. When recombinant proteins were exposed to high temperature (116°C for 30 min), degradation products were detected as smaller sized or reduced intensity signals (S4 Fig.).

These results indicated that recombinant proteins in the PB product are stable for at least 10 months at room temperature under sealed conditions.

### Confocal Microscopy Observation of the Concentrated PB Product

**Fig 5:**
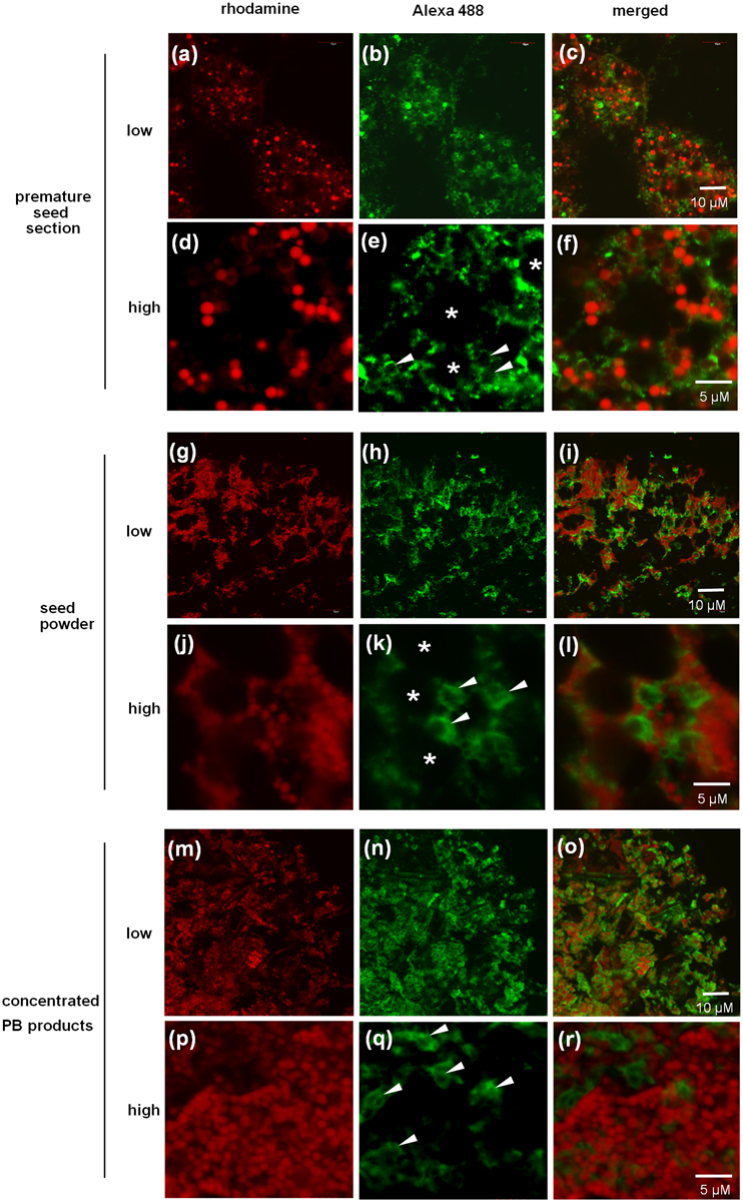
Indirect immunohistochemical analysis of rice cells of premature seed (a–f), seed powder (g–l) and concentrated PB product (m–r). The left panels show PB-I (red), the middle panels show PB-II (green), and the right panels show the merged images. PB-I was detected by rhodamine staining. PB-II was detected by immunohistochemical staining using an anti-GluB antibody. Low and high magnifications are shown. Arrowheads indicate PB-II. Asterisks indicate starch granules.

The organelles in milled seed powder and concentrated PB product were compared by immunostaining and chemical staining with anti-GluB antibody (green) and rhodamine (red) and visualization by confocal microscopy (Fig. 5).

The staining pattern of PB-I (red), PB-II (green) and starch granules (asterisk) was similar between premature rice seeds (Figs. 5a–5l) and milled seed powder, indicating that the components of seed storage organelles were preserved in the cells of milled seed powder. However, in the concentrated PB product, starch granules were not detected and many PB-IIs were deformed, whereas PB-Is showed no morphological changes (Figs. 5m–5r), indicating that PB-Is only were little affected by the concentration process of PBs.

### *In Vitro* Digestion of the Concentrated PB Product with Pepsin

**Fig 6:**
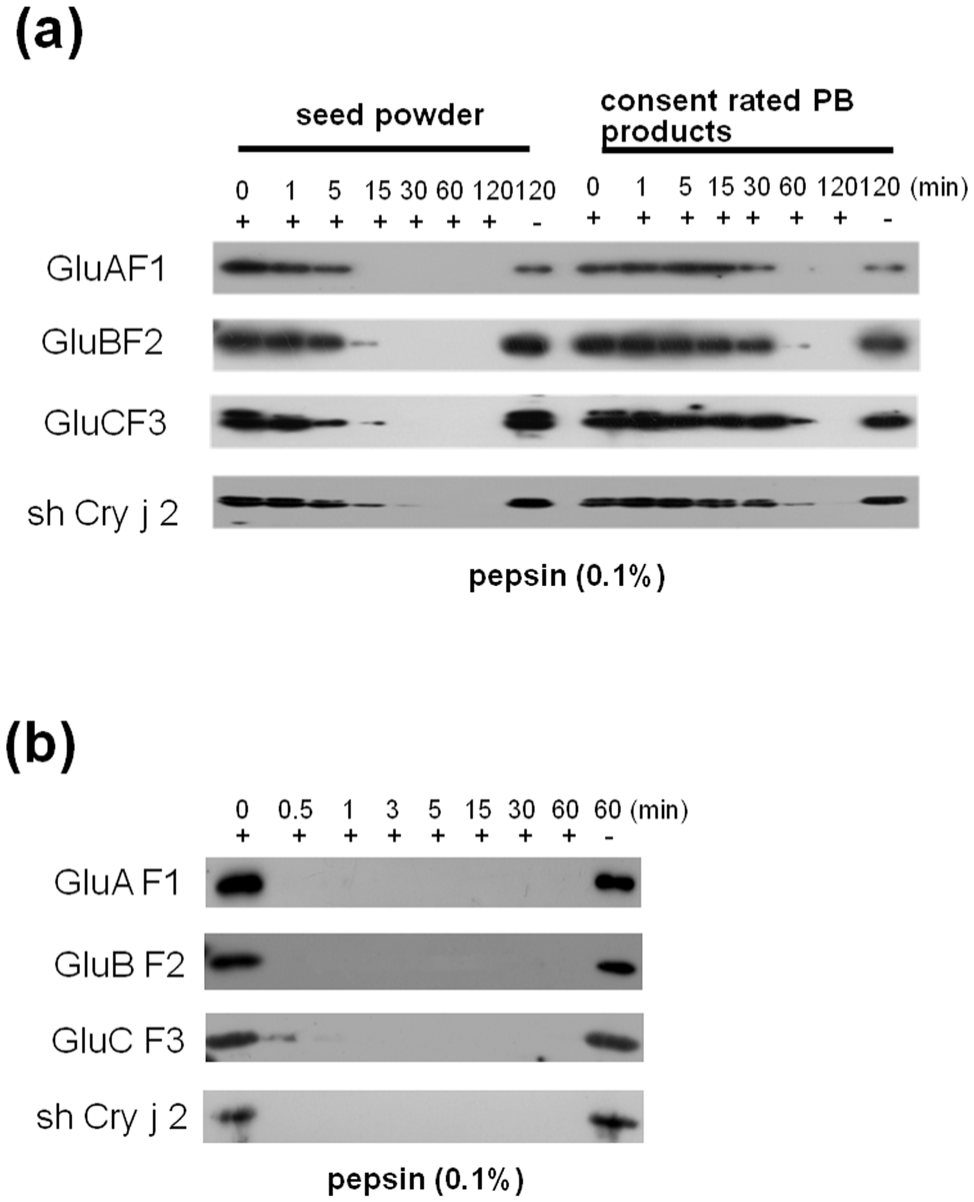
Comparison of resistance to digestive enzyme in rice seed powder and concentrated PB product. (a) Pepsin digestion of seed powder and concentrated PB product for 0, 1, 5, 15, 30, 60, and 120 min at 37°C. Samples consisted of 10 mg of seed powder or 1 mg of concentrated PB products. (b) Pepsin digestion of recombinant protein after extraction from transgenic rice seeds for 0, 0.5, 1, 3, 5, 15, 30, and 60 min.

The digestibility properties of milled seed powder and concentrated PB product were examined in vitro by digestion with simulated gastric juice (0.1% pepsin). To adjust the levels of recombinant proteins, 10 mg of milled seed powder and 1 mg of concentrated PB product were exposed to digestive enzyme solution, and the products were analyzed by immunoblotting. The recombinant proteins in the PB product showed a greater resistance to pepsin than those in the milled seed powder (Fig. 6a).

By contrast, recombinant proteins extracted from the PB product were completely digested with pepsin within 30 to 60 seconds (Fig. 6b).

### Solubility of Total Proteins in Seed Powder and Concentrated PB Product

The major rice seed storage proteins are glutelins (70–80%), globulins (5–10%), and prolamins (20–25%) [14]. In uncooked rice seed, these proteins can be isolated by step-wise extraction based on differences in their physicochemical properties [22]. The 26 kDa α-globulin is soluble in saline buffer. Glutelins can be solubilized with 1% lactic acid after removal of the 26 kDa globulin. Prolamins (cysteine-poor and cysteine-rich prolamins) can be solubilized with 60% propanol containing 5% mercaptoethanol [22]. Because recombinant products form intermolecular disulfide bonds with cysteine-rich prolamins, they were expected to be soluble in lactic acid after removal of cysteine-rich prolamins [23].

**Fig 7:**
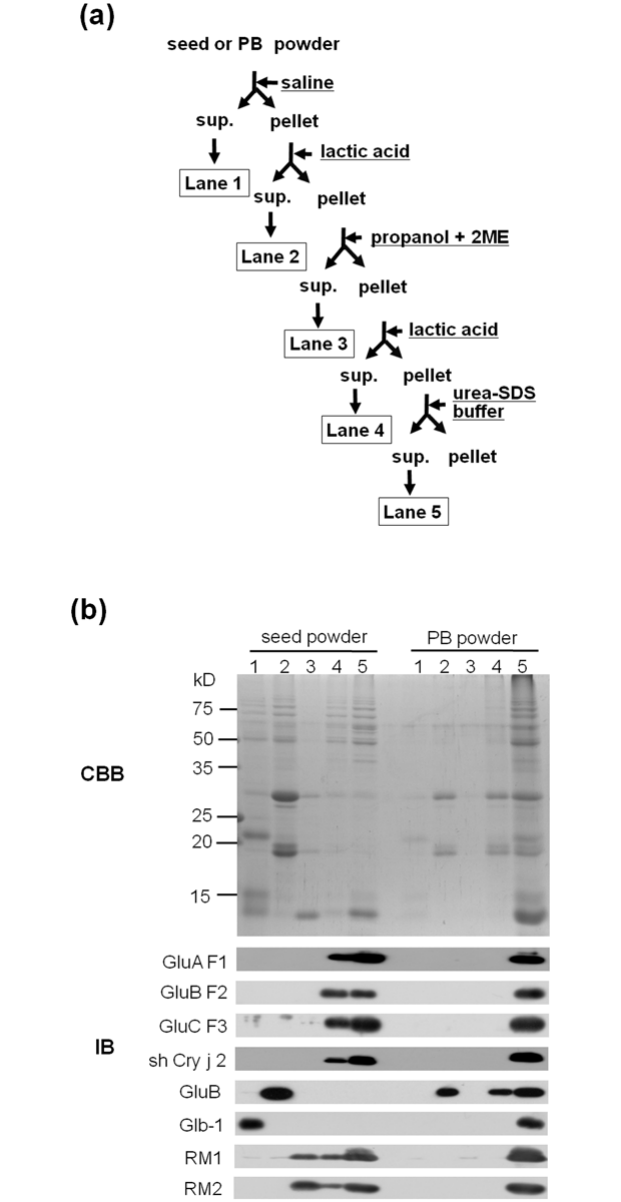
Stepwise extraction of seed proteins from seed powder and concentrated PB product. (a) Flow chart of stepwise extraction. (b) SDS-PAGE (CBB) and immunoblot (IB) analyses are shown. Lane 1, globulin fraction; Lane 2, endogenous glutelin fraction; Lane 3, prolamin fraction; Lane 4, recombinant protein fraction; and Lane 5, pellet fraction.

The solubility of recombinant proteins and endogenous seed proteins was compared between the milled seed powder and the PB product by step-wise extraction, followed by SDS-PAGE and immunoblot analysis of globulins, glutelins, prolamins, transgene products, and the residue (pellet) fraction (Fig. 7).

All components were detected in the milled seed powder in the corresponding fraction. However, in the concentrated PB product, a high proportion of endogenous globulins and prolamins were not detected in the expected fraction, and endogenous glutelins were difficult to extract from the PB product with 1% lactic acid. The fact that most recombinant and endogenous seed storage proteins remained in the residue fraction implies that the process of concentration of PBs changed the physicochemical properties of seed proteins.

### Uptake of Recombinant Protein into Ileum and Jejunum Cells

To determine the location of the uptake of PB-I containing the recombinant proteins in the ileum and jejunum, we generated transgenic rice expressing shuffled Cry j 2 GFP fusion proteins. The fusion proteins were expressed at high levels in the endosperm and accumulated in PB-I, as observed in transgenic rice expressing the shuffled Cry j 2 (S5 Fig.).

In this experiment, the milled seed powder was used for oral administration because in the concentrated PB product, breakdown of GFP during the 90°C digestion with α-amylase resulted in decreased fluorescence intensity.

**Fig 8:**
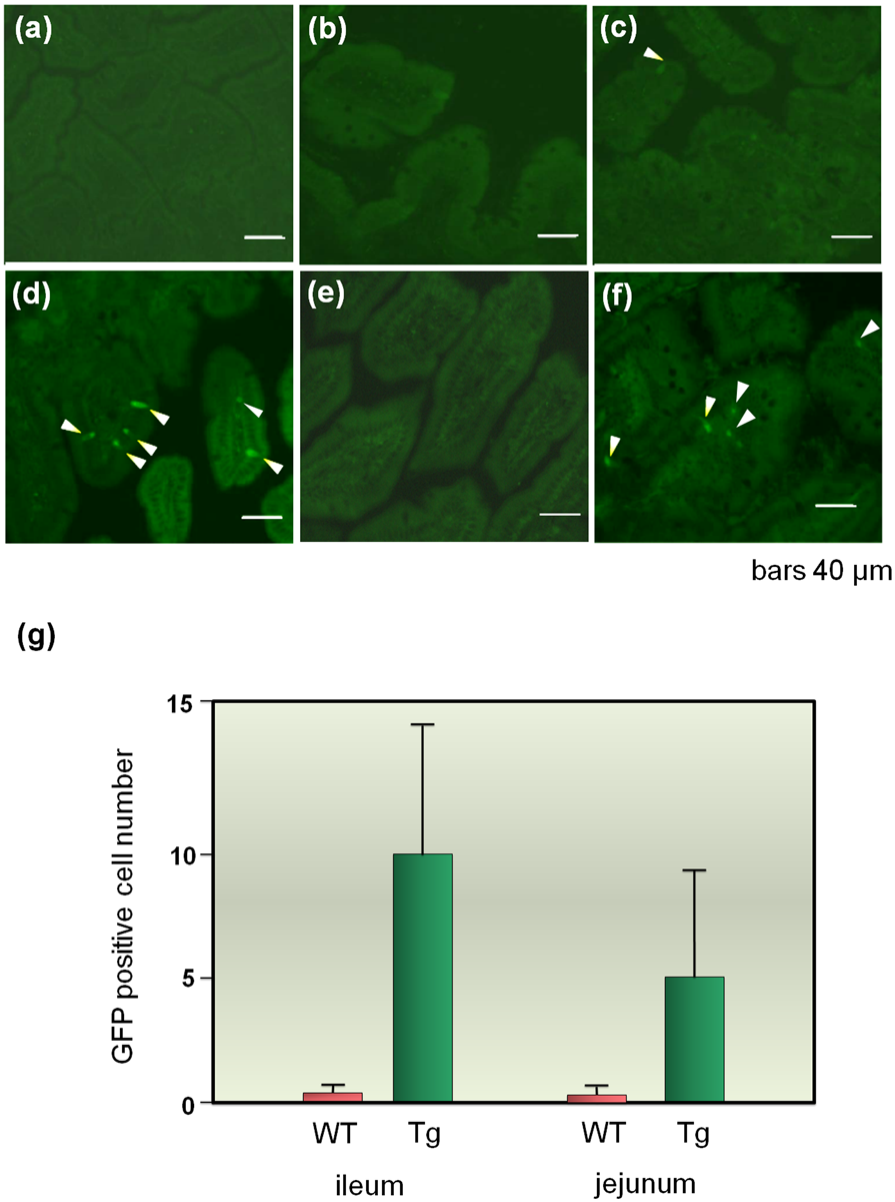
Uptake of antigen (GFP-fused shuffled Cry j 2) by the intestinal tract. (a) Ileum tissues before oral administration of rice seed powder expressing GFP-fused shuffled Cry j 2. (b), (c) and (d) Ileum tissues at 4, 8, and 12 h after oral administration of rice seed powder expressing GFP-fused antigen. (e) Ileum tissues at 12 h after oral administration of non-transgenic rice. (f) Jejunum tissues at 12 h after oral administration of rice seed powder expressing GFP-fused antigen. (g) Average number of GFP spots in 20 regions of a 100 μm^2^ area of ileum and jejunum tissue sections 12 h after oral administration of rice seed powder. WT, wild type; Tg, transgenic rice.

After intragastric administration of the transgenic seed powder in mice, sections of the ileum and jejunum were removed sequentially for observation (Fig. 8).

GFP fluorescence was detected at 8 h after administration and reached a peak at 12 h. Clear GFP fluorescence signals were detected in both ileum and jejunum tissues (Figs. 8d and f). Signals were not detected in these tissues in mice fed non-transgenic rice (Figs. 8a and e). These observations were highly reproducible and were summarized as bar graphs after statistical analysis (Fig. 8g). Particulate antigens such as PB-I are taken up by microfold cells (M-cells) overlying Peyer’s patches (PPs) or directly sampled by dendritic cells (DC) that extend processes into the lumen [24]. However, in the present study, whether the GFP signal was incorporated through M-cells associated with PPs or by direct uptake of DCs in the lamina propria (LP) through dendrites from the lumen remained unclear.

### Oral Administration of PB Concentration Products in a Mouse Model of Japanese Cedar Pollen Allergy

**Fig 9:**
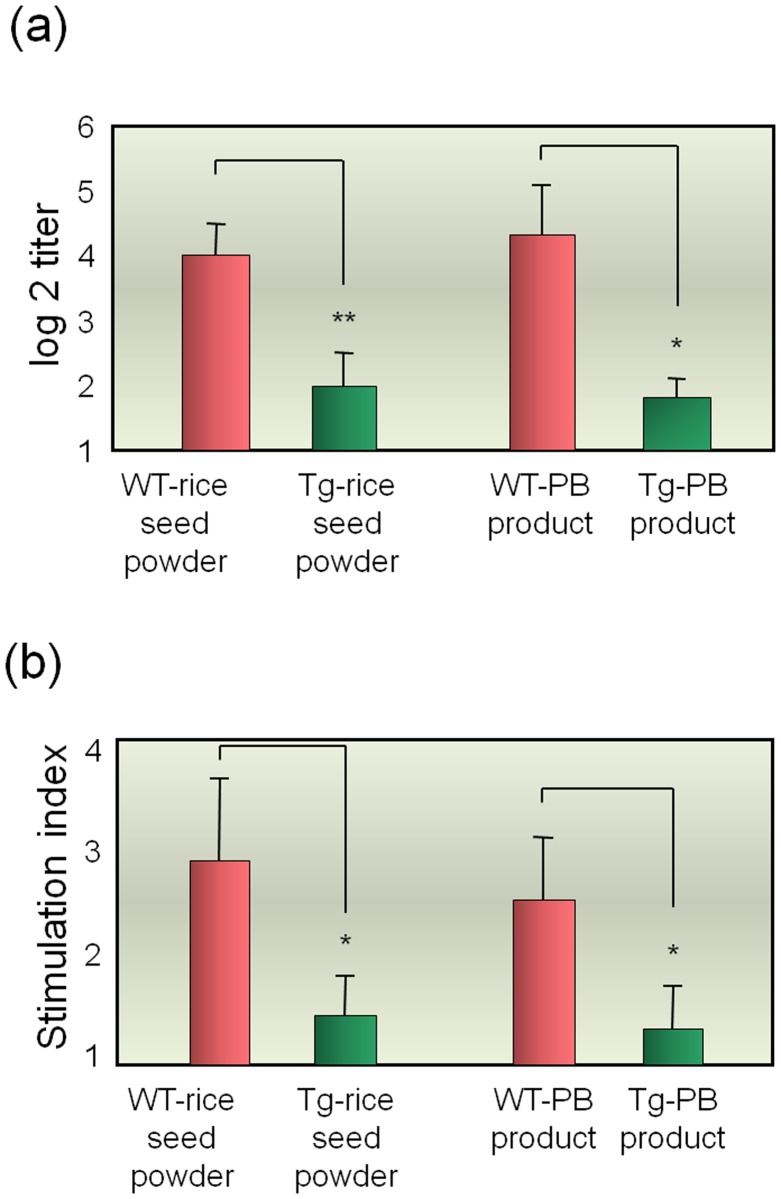
Oral administration of rice seed powder and concentrated PB product. (a) The levels of serum allergen-specific IgE were examined by ELISA. (b) Allergen-specific splenic CD4^+^ T-cell proliferative responses were expressed as stimulation index. Data are expressed as the mean ± standard deviation (*n* = 3 mice per group). ** *P* < 0.01 and * *P* < 0.05 for the group of mice fed Tg-rice seed powder or Tg-PB product in comparison with the group of mice fed WT-rice seed powder or WT-PB product, respectively. WT-rice, wild type rice; Tg-rice, transgenic rice; WT-PB, wild-type concentrated PB; Tg-PB, transgenic concentrated PB.

The induction of immune tolerance by oral administration of the concentrated PB product was examined using a cedar pollen allergy mouse model. Mice were freely fed rice seed powder or PB product for 7 days and then challenged three times with crude allergens of Japanese cedar pollen. The levels of pollen allergen-specific IgE were significantly lower in mice fed transgenic (Tg) rice seed powder and Tg-PB product than in the control group fed wild-type (WT) rice seed powder and WT-PB product (Fig. 9a).

In addition, the allergen-specific T-cell responses were suppressed in mice fed Tg-rice seed powder and Tg-PB product (Fig. 9b). These results indicated the clinical efficacy of concentrated Tg-PB product for the induction of oral immune tolerance to Japanese cedar pollen allergens.

## Discussion

Deconstructed Cry j 1 (fragmentation) and Cry j 2 (shuffling) proteins show lower binding activity to allergen-specific IgE antibodies than the native Cry j proteins [6]. Therefore, oral vaccination with PB product containing these modified Cry j proteins could be an efficient, safe, and convenient system that allows the administration of high doses of antigen required for desensitization without anaphylaxis or pain. We previously reported that oral administration of transgenic rice seed containing the same structurally disrupted Cry j 1 and Cry j 2 induced immune tolerance against these allergens in a mouse model and alleviated clinical symptoms such as sneezing [6]. In the present study, we explored a new formulation consisting of concentrated PB product as an alternative to cooked rice and assessed its advantages regarding distribution and cost effectiveness. The concentrated PB product allowed the delivery of larger amounts of antigen than those provided by milled seed powder or cooked rice. Furthermore, the concentrated PB product can be easily encapsulated similar to conventional pharmaceutical products. The effect of concentrated PB product on the induction of immune tolerance was compared to that of seed powder (Fig. 9).

We showed that the stability of the PB product at ambient temperature was comparable to that of intact seed or milled seed powder (Fig. 3). Recombinant proteins accumulated in rice seed can be stably stored at room temperature for several years [1]. However, in the present study, the stability of the PB product despite the absence of the cell wall and starch was unexpected. Furthermore, the concentrated PB product was more resistant to digestion with artificial gastric juice than the milled seed powder. Considering that the cell walls and carbohydrates, including starch surrounding PBs, play an important role in resistance under harsh conditions and protect against pepsin proteolysis in the stomach, this finding was remarkable [26].

The high stability of the PB product could be attributed to the high temperature concentration process, which involved amylase treatment at 90°C for 1 h. Treatment of rice seed by high temperature, such as that used for the production of cooked rice, alters the physicochemical properties of seed proteins as a result of the formation of protein-protein or protein and starch aggregates [25]. In the present study, most seed storage proteins could not be extracted from the concentrated PB product by step-wise extraction, which differed from the milled seed proteins (Fig. 7). Changes in protein-protein interactions caused by high temperature treatment may account for the high stability and high resistance to enzymatic digestion, irrespective of the absence of the cell wall and starch.

A high resistance to gastrointestinal tract digestion is a desirable characteristic for the induction of oral immune tolerance, because it allows for the delivery of intact recombinant protein antigens to GALT in the small intestine. In the present study, the detection of positive signals for GFP-shuffled Cry j 2 fusion proteins in the in the GALT of the ileum and jejunum (Fig. 8) confirmed the direct uptake of PB-I containing intact recombinant proteins in the gut epithelium and LP. This result is in contrast with the finding that naked recombinant proteins were quickly digested with proteolytic enzymes (simulated gastric juice) within 1 min (Fig. 6b). Particulate material and microbiota mostly enter into Payer’s patches (PP) by M-cell-mediated transcytosis, whereas soluble antigens induce oral tolerance after uptake by DCs in the LP and PP [24, 27]. In addition, protein bodies, as a particulate form, are taken up more efficiently by antibody-producing cells (APCs) than the soluble form of antigens.

Antigen sampling (uptake) occurs at M-cells overlying PPs and at intestinal epithelial cells overlapping the LP on the surface of the intestinal tract. Antigens in the lumen are sometimes captured directly by dendritic cells. Incorporated antigens are processed in APCs, such as dendritic cells and macrophages, and subsequently presented to lymphocytes in PPs and the LP [28, 29]. These APCs are implicated in the induction of immune tolerance through clonal deletion or anergy of specific T-cells and induction of regulatory T-cells. These immune tolerance mechanisms are determined by the tolerizing regimens and depend on dose, duration, and frequency of antigen administration. M-cells can incorporate antigen proteins as well as larger molecules such as bacteria and PBs [30]. The spherical shape of PB-I and its diameter of approximately 1–2 μm [14] allow its direct incorporation into M-cells. M-cell mediated PB-I incorporation was reported in transgenic rice expressing cholera toxin B subunit (CTB), which was characterized using immunohistochemistry with an anti-CTB antibody and Urex europaeus agglutinin as murine M-cell marker [31].

## Conclusion

In the present study, we developed a concentrated PB fraction as an oral tolerogen formulation for allergen-specific immunotherapy. The concentrated PB product showed several advantages such as ease of preparation, high stability at room temperature, high safety, and high efficacy. The design of PB products for oral administration under the guidance of ICH may result in the development of PB product capsules as a new formulation that meets the ICH safety, quality and efficacy guidelines.

## Supporting Information

S1 Fig Immunoblot analysis of endogenous seed storage proteins in the sample described in Fig. 2b. Glutelin B1 (GluB1), 26 kDa globulin (Glb-1), cys-rich prolamin (RM1), and cys-poor prolamin (RM2) are shown.
(TIF)

S2 Fig Residual starch level in the concentrated PB product. (a), Prediction of residual starch in seed powder after incubation in α-amylase solution at 90°C for 0, 5, 20, 40, and 60 min. After incubation, samples were stained with iodine solution. (b), Water insoluble white powder (agarose) containing 15% starch powder (left) and water insoluble powder only (right) were stained with iodine solution. These results indicate that approximate 15% of carbohydrate in the concentrated PB product (Table 1) was not derived from residual starch.
(TIF)

S3 Fig Solubility of α-amylase in water. α-Amylase Termamyl 120L was precipitated with acetone or chloroform. Acetone precipitation can concentrate target proteins without denaturation, whereas chloroform precipitation partially denatures target proteins during the precipitation steps. Precipitates were suspended with 100 μL of water and were divided into supernatant and pellet fractions by centrifugation. An equal volume of urea-SDS buffer was added to the supernatant, and the pellet was dissolved in 200 μL of urea-SDS buffer. S, supernatant; P, pellet;-, without boiling before SDS-PAGE; +, with boiling before SDS-PAGE.
(TIF)

S4 Fig Degradation of recombinant protein by high temperature (116°C for 30 min) in rice seeds. SDS-PAGE and immunoblot analysis of No treatment (NT) and high temperature (116°C) treated-transgenic rice seeds are shown. Degradation products and/or aggregation products were detected in GluAF1, GluBF2, and GluCF3 after high temperature treatment. The levels of shuffled Cry j 2 (sh Cry j 2) were decreased by high temperature treatment, although degradation or aggregation products were not detected.
(TIF)

S5 Fig Development of transgenic rice seed expressing GFP-fused shuffled Cry j 2. (a) Binary vector construct used to express the GFP-fused shuffled Cry j 2 in rice seed tissue. (b) The results of SDS-PAGE (CBB) and immunoblotting (IB) of non-transgenic (wt) and transgenic (Tg) rice seeds are shown. Recombinant proteins were detected using anti-Cry j 2 (sh Cry j 2) and anti-GFP (GFP) antibodies. (c) Confocal microscopy images of premature seeds of transgenic rice. GFP (green), Rhodamine (red), and merged images are shown. GFP-fused recombinant proteins were deposited into PB-I similar to shuffled Cry j 2.
(TIF)
