# Supplementary figures and images for: Concentrated Protein Body Product Derived from Rice Endosperm as an Oral Tolerogen for Allergen-Specific Immunotherapy—A New Mucosal Vaccine Formulation against Japanese Cedar Pollen Allergy
Source: PLoS One. 2015 Mar 16;10(3):e0120209. doi: 10.1371/journal.pone.0120209 (PMC4361645; doi:10.1371/journal.pone.0120209)

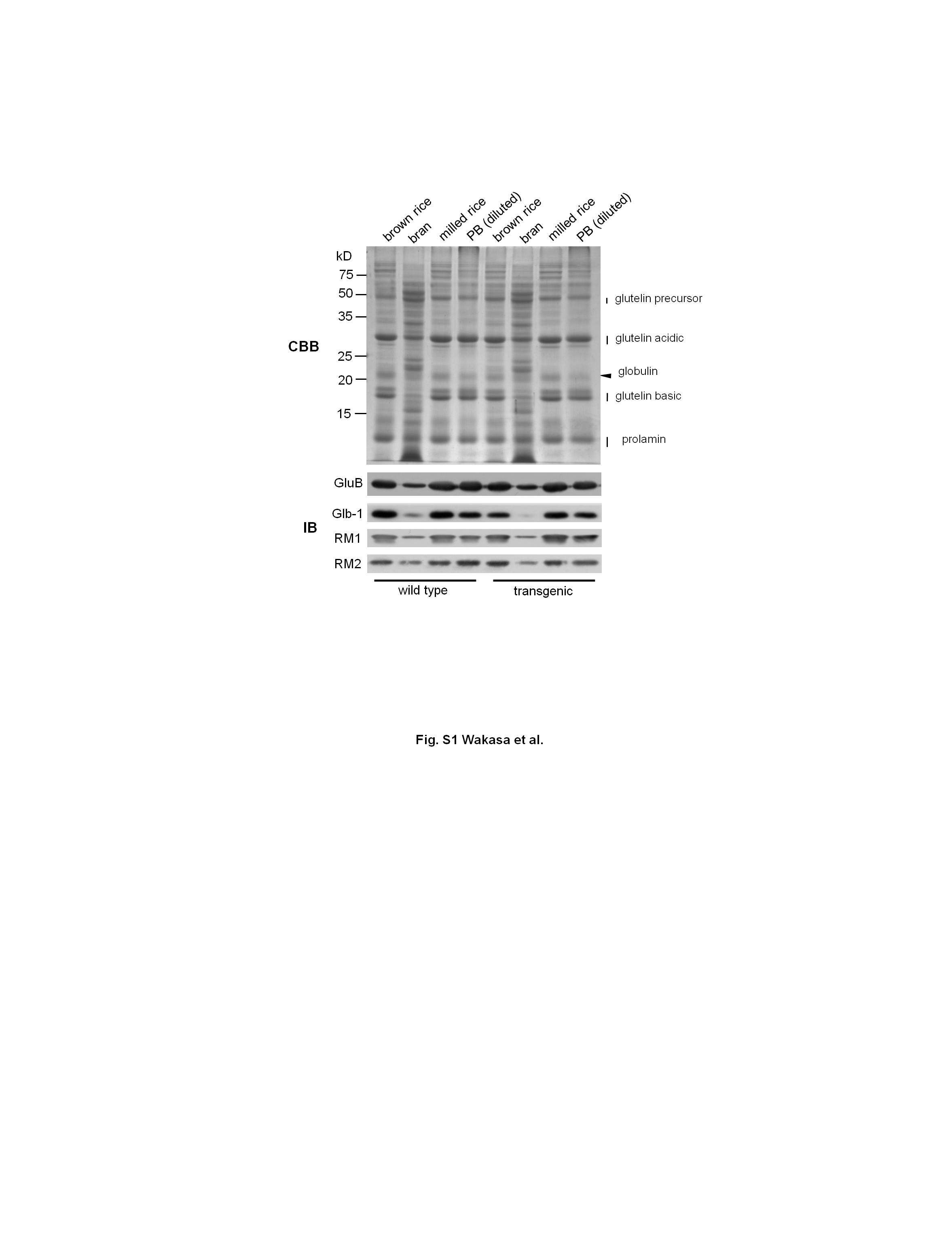

Supplement: S1 Fig — Glutelin B1 (GluB1), 26 kDa globulin (Glb-1), cys-rich prolamin (RM1), and cys-poor prolamin (RM2) are shown. (TIF) [file pone.0120209.s001.tif]

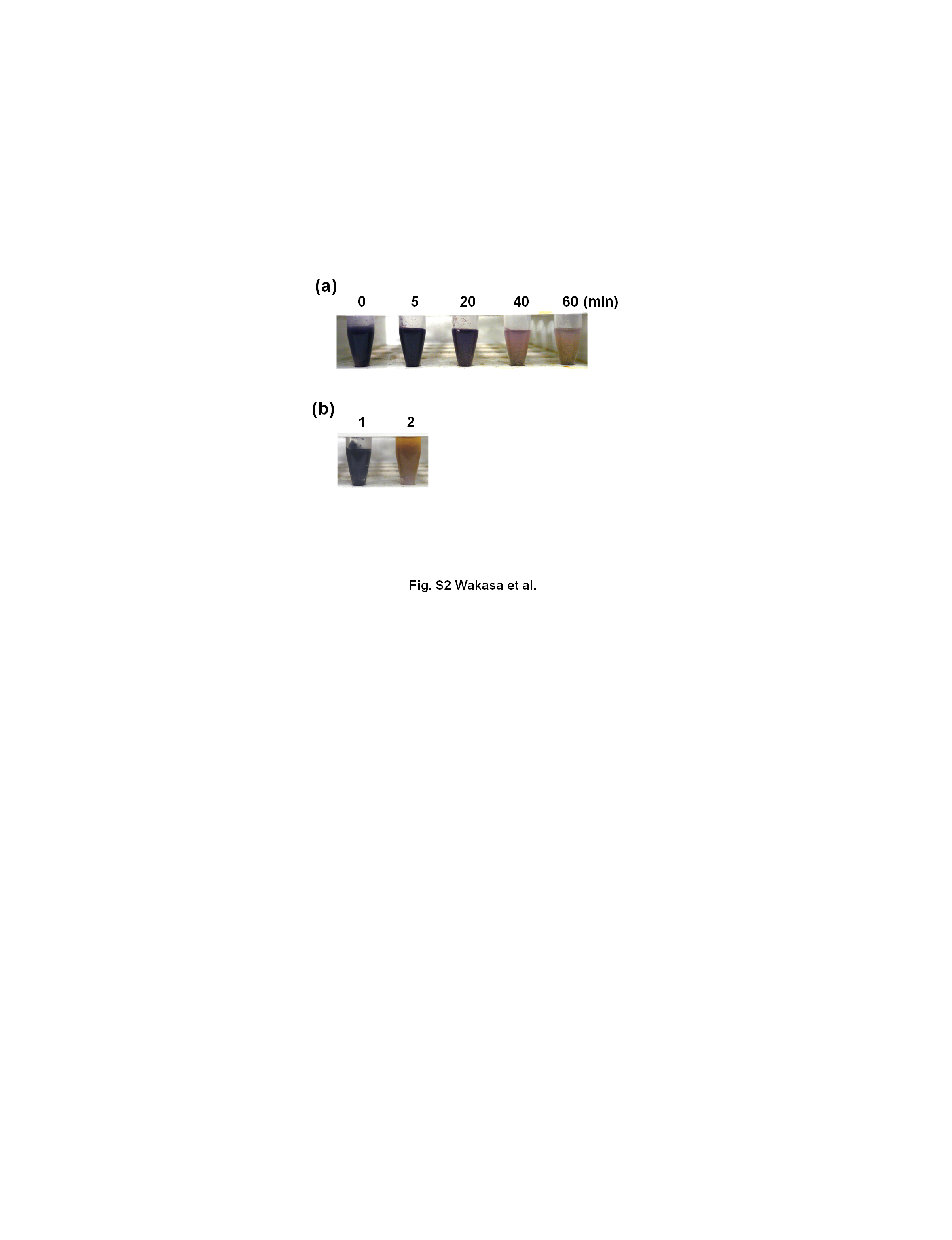

Supplement: S2 Fig — (a), Prediction of residual starch in seed powder after incubation in α-amylase solution at 90°C for 0, 5, 20, 40, and 60 min. After incubation, samples were stained with iodine solution. (b), Water insoluble white powder (agarose) containing 15% starch powder (left) and water insoluble powder only (right) were stained with iodine solution. These results indicate that approximate 15% of carbohydrate in the concentrated PB product (Table 1) was not derived from residual starch. (TIF) [file pone.0120209.s002.tif]

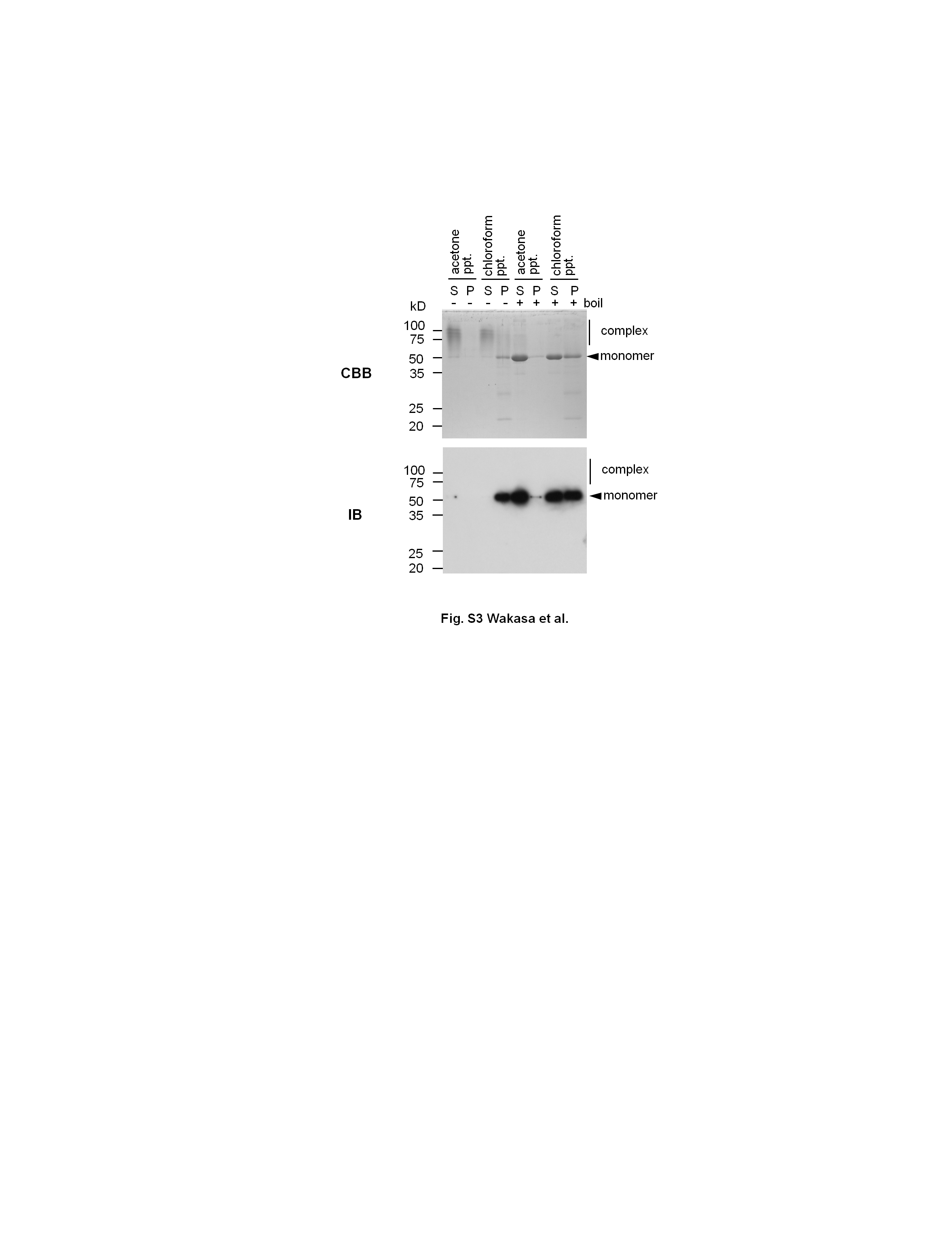

Supplement: S3 Fig — α-Amylase Termamyl 120L was precipitated with acetone or chloroform. Acetone precipitation can concentrate target proteins without denaturation, whereas chloroform precipitation partially denatures target proteins during the precipitation steps. Precipitates were suspended with 100 μL of water and were divided into supernatant and pellet fractions by centrifugation. An equal volume of urea-SDS buffer was added to the supernatant, and the pellet was dissolved in 200 μL of urea-SDS buffer. S, supernatant; P, pellet;-, without boiling before SDS-PAGE; +, with boiling before SDS-PAGE. (TIF) [file pone.0120209.s003.tif]

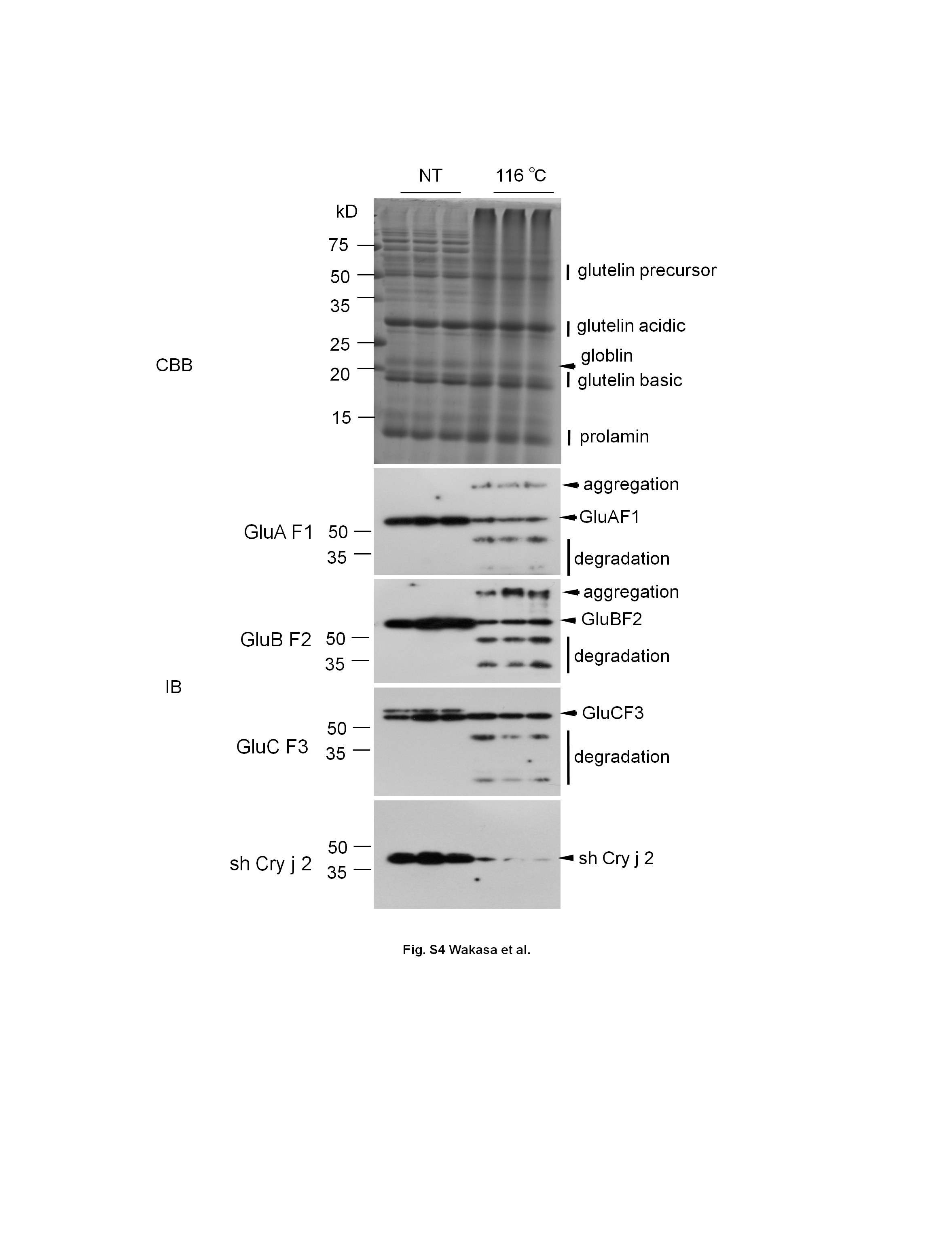

Supplement: S4 Fig — SDS-PAGE and immunoblot analysis of No treatment (NT) and high temperature (116°C) treated-transgenic rice seeds are shown. Degradation products and/or aggregation products were detected in GluAF1, GluBF2, and GluCF3 after high temperature treatment. The levels of shuffled Cry j 2 (sh Cry j 2) were decreased by high temperature treatment, although degradation or aggregation products were not detected. (TIF) [file pone.0120209.s004.tif]

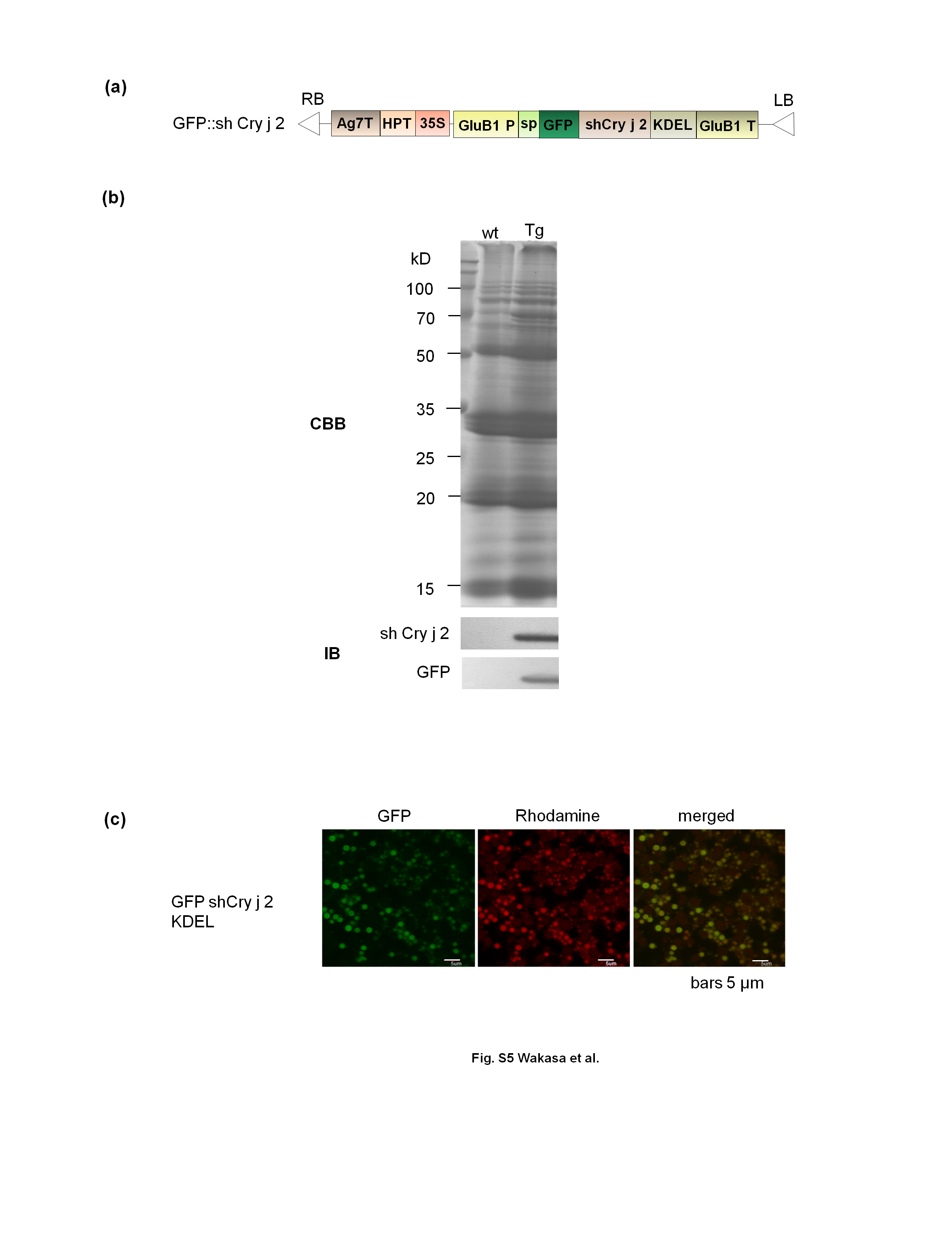

Supplement: S5 Fig — (a) Binary vector construct used to express the GFP-fused shuffled Cry j 2 in rice seed tissue. (b) The results of SDS-PAGE (CBB) and immunoblotting (IB) of non-transgenic (wt) and transgenic (Tg) rice seeds are shown. Recombinant proteins were detected using anti-Cry j 2 (sh Cry j 2) and anti-GFP (GFP) antibodies. (c) Confocal microscopy images of premature seeds of transgenic rice. GFP (green), Rhodamine (red), and merged images are shown. GFP-fused recombinant proteins were deposited into PB-I similar to shuffled Cry j 2. (TIF) [file pone.0120209.s005.tif]
